# Human Leukocyte Antigen (HLA) and Tumor Immunity: A Critical Link in Cancer Immunotherapy

**DOI:** 10.1002/iid3.70363

**Published:** 2026-02-16

**Authors:** Donath Damian

**Affiliations:** ^1^ Department of Biochemistry, Mbeya College of Health and Allied Sciences University of Dar es Salaam Mbeya Tanzania

**Keywords:** cancer progression, human leukocyte antigen (HLA), immune regulation, polymorphism, tumor immunity, tumor immunotherapy

## Abstract

**Background:**

Malignant tumors pose a serious threat to human health and survival, with profound economic consequences worldwide. Human leukocyte antigens (HLAs), encoded by the human major histocompatibility complex, represent one of the most polymorphic genetic systems and play a vital role in immune regulation. This review summarizes the structural and functional characteristics of HLA molecules, their polymorphism and expression in tumor tissues, their involvement in tumor progression and immune responses, and their emerging applications in tumor immunotherapy.

**Methods:**

A thorough literature review was conducted focusing on HLA molecules, their genetic variability in tumor tissues, and their impact on tumor immunity and cellular proliferation. The potential clinical utility of targeting HLA molecules in tumor immunotherapy was also evaluated.

**Results:**

HLA polymorphisms and expression patterns have been closely associated with tumor initiation, progression, and immune modulation. These molecules influence tumor cell growth and regulate antitumor immune responses, either enhancing or suppressing immunity. HLA molecules are therefore critical in shaping the immune system's capacity to detect and eliminate cancer cells.

**Conclusion:**

This review underscores the pivotal role of HLA molecules in cancer immunology. A deeper understanding of HLA‐tumor interactions offers promising avenues for the development of HLA‐based immunotherapies, potentially improving clinical outcomes in cancer treatment.

**Abbreviations**
CTL: cytotoxic T lymphocyte
DC: dendritic cell
HLA‐G: human leukocyte antigen‐G
IFN: interferon
ILT2: immunoglobulin‐like transcript 2
MMP: matrix metalloproteinase
NK: natural killer cells
sHLA‐G: soluble HLA‐G
STAT3: signal transducer and activator of transcription 3
Tregs: regulatory T cells

## 1 Introduction

Malignant tumors continue to pose a serious global health burden, accounting for substantial morbidity, mortality, and socio‐economic impact. The intricacies of tumor development and the capacity of cancer cells to circumvent immune detection present major obstacles in the creation of effective treatments [1]. Central to the immune system's role in recognizing and combating cancer is the human leukocyte antigen (HLA) system. These molecules, encoded by genes within the highly polymorphic major histocompatibility complex (MHC) on Chromosome 6, are integral to immune defense due to their ability to present a broad range of peptides [2, 3].

HLA molecules are essential mediators of antigen presentation to T lymphocytes, playing a critical role in distinguishing self from non‐self, including the identification of tumor‐associated antigens [4]. However, cancer cells often develop mechanisms to escape immune detection, frequently involving disruptions in HLA expression or function [5]. Such immune evasion tactics contribute significantly to cancer progression, as tumors may suppress HLA expression or present altered antigens that go unrecognized by immune cells [6, 7]. This dynamic has sparked increasing interest in leveraging HLA pathways for immunotherapeutic strategies aimed at bolstering anti‐tumor immunity [8].

The extensive polymorphism of HLA genes adds complexity to this immune–tumor interaction. Individual variations in HLA alleles can affect both cancer susceptibility and the patient's response to immunotherapies [9, 10]. Moreover, the variable expression of HLA molecules within tumor environments can facilitate immune escape, further complicating treatment approaches [11].

Given the critical roles of HLA in immune surveillance and cancer development, a comprehensive understanding of their structural characteristics, allele‐specific variations, and functional implications is essential. This review consolidates current research on HLA molecule architecture, the impact of their genetic diversity in tumors, and their involvement in modulating anti‐tumor immune responses. It also explores clinical advancements in HLA‐targeted therapies, including cancer vaccines and adoptive T cell interventions. By delving into the complex interactions between HLA and tumor immunity, this review aims to highlight pathways for improving the precision and efficacy of cancer treatments.

## 2 Structure and Physiological Function of HLA

HLA Class I molecules are generally classified into two types: classical (HLA‐A and HLA‐B) and non‐classical (including HLA‐C, HLA‐E, HLA‐F, and HLA‐G) [12]. Structurally, HLA‐I molecules are composed of a heavy α‐chain and a β2‐microglobulin (β2m) light chain [13]. The α‐chain, encoded by HLA‐A, ‐B, and ‐C genes, consists of three distinct regions: extracellular, transmembrane, and cytoplasmic. The extracellular domain is subdivided into α1, α2, and α3 segments, with the CD8 co‐receptor binding site residing in the α3 domain [14]. The β2m chain, which is not encoded within the HLA complex but by a separate gene, binds non‐covalently to the α3 domain to stabilize the molecule's configuration [15].

In contrast, HLA class II molecules are made up of two transmembrane glycoprotein chains, an α‐ and a β‐chain, encoded by genes near the centromeric region of Chromosome 6 [16]. The primary HLA‐II genes include HLA‐DR, HLA‐DQ, and HLA‐DP, while additional loci such as DMA, DMB, LMP2, LMP7, TAP1, and TAP2 support antigen processing and presentation [17]. Situated between Class I and Class II genes, the Class III region comprises more than 36 genes that produce proteins involved in inflammation and immune signaling, such as complement components (C2, C4, factor B), TNF‐α, TNF‐β, and HSP70 [18, 19].

Among non‐classical HLA‐I molecules, HLA‐G and HLA‐E have attracted growing interest due to their roles in immune modulation [20]. Found at Chromosome 6p21.3, HLA‐G plays a key part in maternal–fetal tolerance by preventing maternal natural killer (NK) cells from targeting the fetus [21]. It is secreted by several cell types during early pregnancy, including cytotrophoblasts, amniotic epithelial cells, and erythroid precursors, and reduced expression has been linked to conditions such as pre‐eclampsia and recurrent pregnancy loss [22]. Meanwhile, HLA‐E is expressed on endothelial cells, various immune cells, and trophoblasts at the fetal interface [23]. It interacts with C‐type lectin receptors, including inhibitory receptors like CD94/NKG2A and activating receptors such as CD94/NKG2C, which regulate NK cell activity [24].

HLA molecules perform two primary functions: targeting antigens and enabling immune recognition [25]. The specific peptide sequence presented by HLA‐I molecules determines their antigenic profile and T cell specificity [26]. This process underpins the collaborative nature of the immune response, where macrophages present processed antigens to helper T cells, which then activate B cells for antibody production [27]. The efficacy of this mechanism depends on genetic compatibility between antigen‐presenting cells (APCs) and T cells [28].

Beyond immune surveillance, HLAs are also crucial in transfusion reactions and organ transplantation. Many febrile non‐hemolytic transfusion reactions are caused by anti‐HLA antibodies, especially in patients with prior transfusions [29]. In organ and bone marrow transplantation, HLA compatibility is a decisive factor in graft survival and the prevention of graft‐versus‐host disease (GVHD) [30].

Due to their extreme polymorphism, HLA genes are also widely used in forensic science for individual identification [31]. However, this genetic variability complicates donor‐recipient matching and the identification of disease‐linked alleles. Over 60 diseases have been associated with HLA, including ankylosing spondylitis, where more than 91% of affected white patients carry the HLA‐B27 allele [32]. Associations have also been observed in multiple autoimmune and inflammatory conditions, including Hodgkin's lymphoma and Behçet's disease [33].

**Figure 1:**
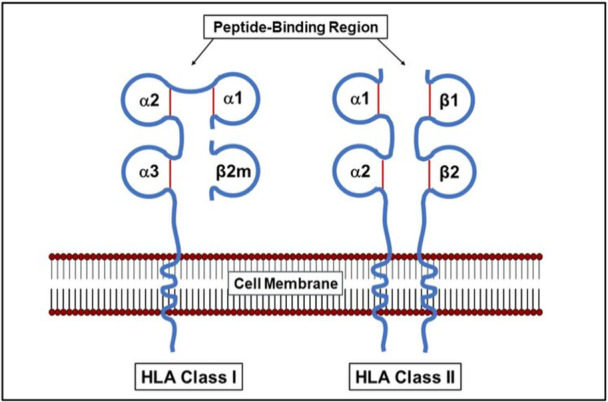
Structure of HLA Class I and Class II molecules. HLA Class I molecules comprise a polymorphic heavy alpha chain paired with a non‐polymorphic light chain known as beta2‐microglobulin (β2m). In contrast, HLA Class II molecules consist of two polymorphic chains: alpha and beta (NCBI Bookshelf).

Overall, the structural and functional properties of HLA molecules are not only central to immune defense but also carry broad biomedical significance, from understanding disease pathogenesis to advancing personalized medicine (Figure 1).

## 3 The Role of HLA Polymorphism and Expression in Tumor Tissues

The association between HLA genetic variability and cancer susceptibility differs considerably across ethnic groups, and findings often vary between populations (see Table 1). For example, in Spanish individuals with liver conditions, the HLA‐DR11 allele was significantly more common among hepatitis C virus (HCV) carriers compared to those with terminal liver disease or liver malignancies [34]. Similarly, the HLA‐B18 allele appeared more frequently in patients with hepatocellular carcinoma (HCC) but was absent in those carrying HCV [35]. Another study reported increased prevalence of the HLA‐A4 allele in HCC patients compared to HCV‐infected individuals [36]. Among Yugoslav patients with HBsAg‐positive hepatoma, the HLA‐B15 antigen was more prevalent than in individuals with chronic liver conditions or HBV carriers [37]. In an Italian cohort with HCC, alleles like CW7, B8, and DR3 were found at elevated frequencies [38].

**Table 1:** Associations between HLA variants and various cancer types.

Cancer type	Associated HLA alleles/Haplotypes	References
Hepatocellular carcinoma	HLA‐DR11, HLA‐B18, HLA‐A4, HLA‐B15, HLA‐CW7, HLA‐B8, HLA‐DR3	[62, 63, 64, 65, 66]
	HLA‐DRB107, HLA‐DRB104, HLA‐DQB102, HLA‐DRB1101	[67, 68, 69, 70]
Ovarian cancer	HLA‐A1, HLA‐A2, HLA‐B5, HLA‐DRB103, HLA‐DRB104	[71, 72, 73, 74, 75, 76]
Cervical cancer	HLA‐CW3, HLA‐DRB1*0301, HLA‐DQA1*0501, HLA‐DQB1*0201, HLA‐DQA1*0101, HLA‐DRB1*1001, HLA‐DQB1*0501	[77, 78, 79, 80]
	HLA‐DRB1*04, HLA‐DRB1*07, HLA‐DRB1*11, HLA‐DRB1*15, HLA‐DRB1*1501	[81, 82, 83]
Glioma	HLA‐DQA1*0102	[84]
Kaposi's sarcoma	Not specified	[85]
Oral tumors/HNSCC	HLA‐CW7, HLA‐DRB1*1104, HLA‐DRB1*1302, HLA‐DQA1*0302, HLA‐DQB1*0604, HLA‐B, HLA‐DRB1*13	[86, 87, 88, 89]

In Egyptian HCC patients, certain alleles such as DRB107, DRB104, and DQB102 were significantly overrepresented, pointing toward their potential role as genetic risk factors. In contrast, alleles like DQB1 and DRB115 were less frequent, indicating a possible protective effect [39]. A study by [40] further highlighted the significance of DRB1 and DQB1 haplotypes, showing that DQB1 may aid in clearing HCV naturally, while DRB1 appeared to increase the likelihood of developing HCV‐induced HCC.

In the context of ovarian cancer, increased frequencies of HLA‐A1 and HLA‐A2 alleles were observed in patients compared to healthy individuals, while HLA‐A3 was less common [41]. Haplotypes such as HLA‐A2:B8, along with combinations like A2, B5, DRB1, and CW3, and class II variants including DRB1, DQA1, and DQB1, were found more often in patients, implying a potential contribution to disease development [42]. In cervical cancer, particularly HPV16‐positive squamous cell carcinoma, alleles such as HLA‐DRB104, DRB107, DRB111, and DRB115 were associated with higher risk [43]. In contrast, HLA‐DRB11402 and HLA‐A02 were linked to lower incidence, while DRB11501 and DQA10102 were connected to increased susceptibility [44].

HLA polymorphisms also play key roles in other malignancies. For instance, HLA‐DRB114 has been identified as a risk factor for glioma [45], and specific haplotypes such as HLA‐CW7, DRB11104, DRB11302, DQA10302, and DQB10604 are linked to Kaposi's sarcoma [46]. In head and neck squamous cell carcinoma, the HLA‐B35 allele has shown protective effects by suppressing metastasis, whereas HLA‐B40 and DRB113 were associated with enhanced tumor development [47].

The expression levels of non‐classical HLA molecules, particularly HLA‐G, have been correlated with cancer progression in several tumors. In breast cancer, higher HLA‐G expression was associated with larger tumor size, lymph node involvement, and advanced TNM stage, and was linked to worse overall survival [48]. Additionally, increased levels of soluble HLA‐G (sHLA‐G) were positively associated with the number of regulatory T cells (Tregs) (CD4+CD25^highFoxp3^+) in affected patients [49]. In advanced ovarian cancer, overexpression of both HLA‐G mRNA and protein corresponded with poorer clinical outcomes, suggesting its utility as a biomarker of disease severity [50].

In non‐small cell lung cancer (NSCLC), HLA‐G overexpression was significantly related to disease stage, lymphatic spread, and immune alterations, including elevated interleukin‐10 (IL‐10) and loss of classical HLA‐I genes [51]. In renal cancer, HLA‐G levels were notably higher in tumor samples than in adjacent healthy tissues, with its expression being more prominent than that of other HLA types [52]. Similar trends were found in esophageal cancer, HCC, and colorectal cancer, where high HLA‐G expression was linked to poorer prognosis, reduced survival, and increased recurrence [53, 54]. Elevated sHLA‐G levels in these cancers have been proposed as potential diagnostic indicators [55, 56].

**Figure 2:**
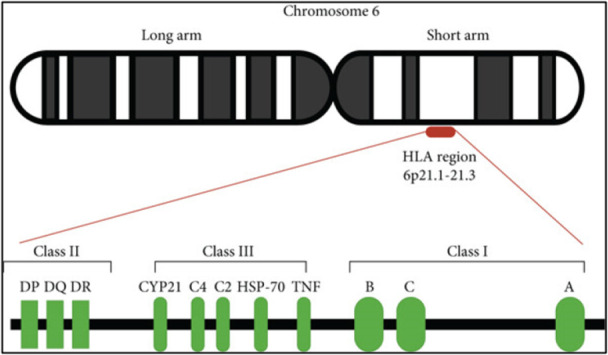
The human leukocyte antigen (HLA) complex is located on the short arm of Chromosome 6 at position 6p21, covering roughly 4000 kilobases of genomic DNA. This region contains genes organized into three primary classes: Class I (including HLA‐A, HLA‐B, and HLA‐C), Class II (comprising HLA‐DP, HLA‐DQ, and HLA‐DR), and Class III, which encodes a variety of immune‐related proteins such as components of the complement cascade, 21‐hydroxylase, heat shock proteins, and tumor necrosis factors (TNFs).

Moreover, in cervical cancer, both HLA‐E and HLA‐G expression levels were increased when compared to tissues from chronic cervicitis or cervical intraepithelial neoplasia (CIN). Their expression correlated with clinical parameters such as tumor differentiation, CIN grade, TNM classification, and HPV infection [57]. In gastric cancer, higher levels of sHLA‐E and HLA‐G were associated with advanced disease stages [58]. In liver cancer, upregulated HLA‐E expression was linked to tumor recurrence [59]. Similarly, in breast cancer, both the gene frequency and soluble levels of HLA‐E were elevated in patients, pointing toward its role in disease risk and progression [60, 61] (Figure 2).

Where alleles are listed with asterisks (e.g., DRB1*0301), this reflects high‐resolution HLA typing [62, 63, 64].

## 4 HLA Molecules and Their Role in Immune Surveillance of Tumors

**Figure 3:**
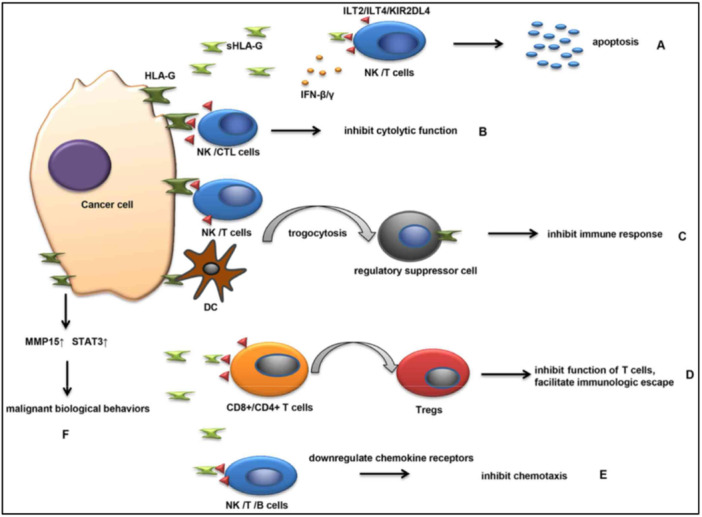
Mechanisms by which HLA‐G and soluble HLA‐G (sHLA‐G) contribute to tumor immune evasion and progression. (A) Apoptosis, (B) Cytolytic function inhibition, (C) Immune response inhibition, (D) T cells function inhibition, (E) Inhibition of chemotaxis, (F) Malignant biological behaviors.

HLA Class I molecules play a [65] fundamental role in presenting intracellular peptides on the surface of cells, enabling the immune system—particularly CD8+ cytotoxic T lymphocytes (CTLs)—to recognize and eliminate [66, 67, 68, 69]; cells expressing tumor‐specific neoantigens [70, 71, 72, 73, 74, 75, 76, 77] (see Figure 3). Effective expression of HLA‐I on tumor cells is essential for their detection and destruction by CTLs [78, 79, 80, 81, 82, 83, 84, 85]. However, tumor cells frequently acquire mutations or epigenetic alterations that result in reduced or lost HLA‐I expression [86, 87, 88], allowing them to evade immune responses. This downregulation disrupts [89, 90] CTL functions, including secretion of perforin, granzyme B, IFN‐γ, and TNF‐α, weakening anti‐tumor activity [91, 92].

Restoring HLA‐I expression in pancreatic ductal adenocarcinoma has been shown to enhance antigen presentation, thereby improving CTL‐mediated responses and slowing tumor growth [93]. On the other hand, activation of the Wnt/β‐catenin signaling pathway has been shown to suppress HLA‐I expression, reducing CTL activity against tumors [94]. Tumors also use epigenetic silencers like Polycomb Repressive Complex 2 (PRC2) to block MHC‐I antigen processing pathways [95]. Inhibition of EZH1 and EZH2, core components of PRC2, can reverse this silencing, leading to restored T cell immunity [96].

Radiation therapy has also been reported to upregulate HLA‐I expression on tumor cells, making them more susceptible to CTL‐mediated killing [97]. Alongside adaptive immunity, NK cells serve as key players in the innate immune defense against tumors [98]. Unlike CTLs, NK cells do not require MHC restriction and can recognize and kill tumor cells that lack HLA‐I expression [99]. The activity of NK cells is influenced by non‐classical HLA molecules, such as MICA, which binds to the NKG2D receptor and initiates NK cell activation [100].

MICA gene polymorphisms have been linked to cancer susceptibility in conditions like gastric cancer and oral squamous cell carcinoma [101, 102]. MICA+ leukemia cells, for example, are more prone to NK‐mediated cytotoxicity compared to MICA– counterparts [103]. In cancers such as lung, cervical, and colorectal, MICA expression has been associated with stronger NKG2D‐mediated immune responses [104]. However, some tumors, like SKOV3 ovarian cancer cells, despite expressing MICA, do not effectively engage NKG2D, in contrast to HeLa cells, which trigger a more robust immune response [105].

Large‐scale transcriptomic analysis from TCGA and GTEx databases revealed that MICA expression is generally higher in normal tissues compared to tumor tissues across various cancer types, including breast and colon cancers [106]. While most tumor cells express MHC Class I, the expression of MHC Class II molecules is typically limited [107]. Historically, research has focused on MHC‐I‐restricted tumor antigens, but increasing evidence suggests a significant role for MHC‐II molecules in tumor immunity [108]. For instance, Toll‐like receptor 2 (TLR2) activation can suppress MHC‐II expression in microglial cells, impairing CD4+T cell activation and contributing to immune evasion [109].

In chronic myeloid leukemia (CML), interferon‐γ can enhance MHC‐II expression, though this can be counteracted by JAK1/2 inhibitors like ruxolitinib [110]. In breast cancer, tumor cell expression of MHC‐II supports the activation of CD4+ helper T cells, which in turn augment CD8+T cell responses, inhibiting tumor growth [111]. Studies in melanoma, colon, and breast cancer show that CD4+T cells can directly recognize tumor cells, reinforcing the potential importance of MHC‐II in tumor antigen recognition [112]. This opens the door for MHC‐II‐targeted immunotherapies.

Another important immune modulator is HLA‐G, a non‐classical HLA‐I molecule secreted by some tumor cells. HLA‐G suppresses both NK cell and CTL activity, thereby facilitating immune escape [113]. The process by which HLA‐G supports tumor immune evasion can be conceptualized in three phases: elimination, equilibrium, and escape [114]. In the elimination stage, HLA‐G interacts with ILT2 and ILT4 receptors on immune cells, blocking their cytotoxic actions [115]. During the equilibrium phase, HLA‐G levels are moderate but still impair immune activation and promote the generation of Tregs [116]. In the escape phase, factors like hypoxia stimulate HLA‐G overexpression, resulting in widespread immune suppression and tumor progression [117].

Finally, HLA‐E is another non‐classical molecule that regulates immune responses by binding to the inhibitory NKG2A receptor on both NK and CD8+T cells [118]. This interaction activates downstream signaling that reduces immune cell activity, aiding in tumor immune evasion and promoting disease progression [119].

HLA‐G and its soluble form, sHLA‐G, contribute to tumor development through several immunosuppressive mechanisms:

A. sHLA‐G secreted by tumor cells binds to inhibitory receptors on NK cells and T lymphocytes, inducing their apoptosis and reducing immune surveillance.
B. Membrane‐bound HLA‐G on tumor cells interacts directly with receptors on activated NK and CTLs, leading to functional inhibition of these immune cells.
C. Tumor cells can transfer membrane fragments containing HLA‐G to NK cells, dendritic cells (DCs), or T cells through direct contact. These recipient cells temporarily adopt regulatory phenotypes, suppressing immune responses.
D. In the presence of sHLA‐G, CD4+ and CD8+T cells lose their responsiveness to antigens and differentiate into Tregs, which suppress effector T cell activity and promote immune tolerance.
E. sHLA‐G impairs the chemotactic capabilities of NK, T, and B cells by interacting with the ILT2 receptor, downregulating chemokine receptor expression, and preventing immune cells from migrating to tumor sites.
F. HLA‐G may also enhance tumor invasiveness and metastatic potential by upregulating matrix metalloproteinases (MMPs) and activating signal transducer and activator of transcription 3 (STAT3) signaling pathways.

## 5 Therapeutic Targeting of HLA in Tumor Immunotherapy

**Figure 4:**
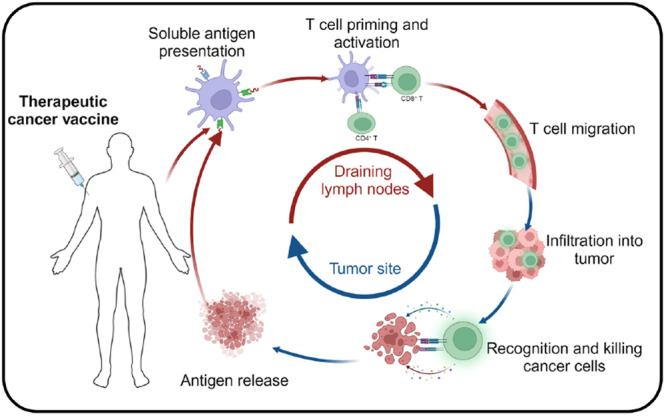
Mechanism of cancer vaccine action in vivo.

A key challenge in early T cell‐mediated immune responses is the weak interaction between T‐cell receptors (TCRs) and peptide‐MHC complexes when the peptides have low binding affinity. These unstable interactions can result in poor immune recognition, allowing tumor cells to evade immune destruction [120] (see Figure 4). Therefore, the precise identification and selection of tumor‐derived peptides that bind strongly to MHC molecules is vital for the development of effective and safe cancer vaccines [121].

Mass spectrometry has become an indispensable tool for detecting tumor‐specific neoantigens directly from cancer tissues or cells, aiding in the design of personalized cancer vaccines [122]. Additionally, the use of oncolytic viruses has been shown to stimulate tumor cells to express novel MHC class I ligands, thereby enhancing CD8+T cell responses and promoting immune‐mediated tumor killing [123].

An innovative strategy involving a peptide‐MHC class I‐IgG fusion protein has demonstrated success in preclinical models by targeting lung cancer cells and activating CD8+T cells in vivo, resulting in suppressed tumor growth [124]. These strategies aim to develop vaccines that harness tumor‐specific neoantigens presented by MHC molecules, thereby tailoring neoantigen‐based immunotherapy to individual patients [125].

Further research has investigated the use of DNA vaccines encoding tumor neoantigens in animal models. These vaccines successfully elicited MHC Class I‐restricted CD8+T cell responses and showed strong anti‐tumor efficacy. The neoantigen‐specific T cells generated in response to the vaccine were able to recognize and eliminate tumor cells both in vitro and in vivo [126].

Although most immunotherapy efforts have focused on MHC Class I‐restricted antigens, increasing evidence highlights the importance of MHC Class II‐restricted peptides in tumor immunity [127]. In fact, CD4+T cells targeting MHC‐II‐bound neoantigens appear to exert stronger selective pressure on tumors than CD8+T cells, indicating that helper T cell responses play a central role in tumor immune surveillance [128]. Recent clinical studies have identified MHC class II‐restricted neoantigens in tumor‐infiltrating lymphocytes (TILs) from patients with metastatic cholangiocarcinoma, and reinfusion of CD4+T cells recognizing these neoantigens showed promising therapeutic potential [129].

Immune checkpoint inhibitors (ICIs) have become a breakthrough in cancer immunotherapy by enhancing T cell responses against tumors [130]. However, some tumors, such as pancreatic ductal adenocarcinoma, evade immune detection through a mechanism involving selective autophagy of MHC Class I molecules, leading to reduced antigen presentation [131]. Research has shown that blocking autophagy or lysosomal degradation can restore MHC‐I expression on tumor cells, thus enhancing the immune system's ability to recognize and destroy them, especially when combined with ICIs [132].

In endometrial cancer, low levels of MHC Class I expression have been identified as a potential reason for resistance to checkpoint blockade therapy, emphasizing the importance of restoring MHC‐I expression to improve treatment outcomes [133, 134]. Other studies have found that CXCL14, a chemokine, can suppress HPV‐positive cervical cancer by upregulating MHC class I expression and promoting CD8+T‐cell‐mediated immune responses [135, 136]. Collectively, these findings underscore the therapeutic value of enhancing MHC class I expression to improve the efficacy of immunotherapies, especially ICIs, across various cancer types.

Once introduced into the body, tumor antigens—delivered in various forms—are taken up by specialized APCs such as DCs. These antigens are processed within the APCs and presented on their surface via MHC molecules. The antigen‐MHC complexes are recognized by TCRs on the surface of antigen‐specific T cells, triggering their activation. Activated T cells then target and eliminate tumor cells in a precise, sustained, and immune‐specific manner, ultimately suppressing tumor growth and progression.

## 6 The Future of HLA‐Based Cancer Immunotherapy

This review has highlighted the crucial connection between HLA polymorphisms and cancer progression, with a particular focus on the central role of HLA molecules in modulating immune responses against tumors. Effective immunotherapy relies on the coordinated activity of immune cells such as NK cells, CD4+ helper T cells, and CD8+ cytotoxic T lymphocytes. Although advances in cancer vaccines and adoptive T‐cell therapies targeting MHC‐restricted antigens have been encouraging, these strategies still face limitations in completely eliminating tumors, and their therapeutic efficacy remains under active investigation and refinement.

HLA gene variants have been implicated in the development and immune evasion of several malignancies, including lung, liver, and gastric cancers. However, due to the extensive genetic variability of HLA alleles across individuals and populations, research findings often lack consistency and reproducibility. Addressing this issue will require the integration of high‐throughput genomic data and advanced computational methods to unravel complex HLA‐tumor interactions and generate more reliable insights.

Emerging evidence also points to the influence of HLA genotypes on patient response to ICIs. This suggests that HLA typing could serve as a predictive biomarker, enabling more personalized and targeted cancer immunotherapies. Future directions should focus on incorporating HLA profiling into treatment planning, allowing clinicians to match therapies with each patient's unique immunogenetic landscape.

## 7 Conclusion

HLA diversity plays a critical role in shaping the immune system's ability to recognize and combat cancer cells. While immunotherapies, including MHC‐restricted cancer vaccines and adoptive T‐cell approaches, have demonstrated substantial promise, they are not yet capable of achieving complete tumor clearance in all cases. The complex and highly polymorphic nature of the HLA system complicates efforts to generalize findings across different cancer types and patient populations.

Nonetheless, growing evidence supports the integration of HLA‐related biomarkers into the design of personalized immunotherapies, particularly in enhancing the effectiveness of ICIs. Continued research, especially leveraging big data analytics and precision medicine, will be vital in advancing the development of more effective and individualized cancer treatments. By deepening our understanding of HLA's role in tumor immunology, we move closer to achieving long‐term success in cancer immunotherapy.

## Author Contributions

The author solely conceived and designed the study, developed the methodology, conducted the literature review and data collection, performed the analysis and interpretation of findings, and drafted and critically revised the manuscript. The author approved the final version of the manuscript and takes full responsibility for the integrity and accuracy of the work.

## Funding

The author received no specific funding for this work.

## Ethics Statement

The author has nothing to report.

## Consent

The author has nothing to report.

## Conflicts of Interest

The author declares no conflicts of interest.
